# “We’re one small piece of the puzzle”: evaluating the impact of short-term funding for tier two weight management services

**DOI:** 10.3389/fpubh.2024.1381079

**Published:** 2024-05-22

**Authors:** Jordan D. Beaumont, Elysa Ioannou, Krishna Harish, Nnedinma Elewendu, Nicola Corrigan, Lucie Nield

**Affiliations:** ^1^College of Business, Technology and Engineering, Sheffield Hallam University, Sheffield, United Kingdom; ^2^Sport and Physical Activity Research Centre, Sheffield Hallam University, Sheffield, United Kingdom; ^3^College of Social Sciences and Arts, Sheffield Hallam University, Sheffield, United Kingdom; ^4^Office for Health Improvement and Disparities, Department of Health and Social Care, Blenheim House, Leeds, United Kingdom

**Keywords:** obesity, public health, service evaluation, government funding, weight management

## Abstract

**Introduction:**

Overweight and obesity are a global health epidemic and many attempts have been made to address the rising prevalence. In March 2021 the UK government announced £100 million of additional funding for weight management provisions. Of this, £30.5 million was split across local authorities in England to support the expansion of tier two behavioural weight management services for adults. The present work aimed to explore how this funding was used within the Yorkshire and Humber region to consolidate learning, collate best practice, and provide recommendations for future funding use.

**Method:**

One-hour semi-structured interviews were conducted with 11 weight management service commissioners representing 9 of the 15 local authorities in the region. Interviews were recorded, transcribed and analysed using an established health inequality framework. From this, recommendations were co-developed with the commissioner group to establish best practice for future funding use.

**Results:**

Commissioners recognised that targeted weight management services were only one small piece of the puzzle for effectively managing obesity. Therefore, recommendations include targeting underserved communities, focussing on early prevention, addressing weight management in a whole systems context, and embracing innovative and holistic approaches to weight management.

**Discussion:**

Current short-term funding and restrictive commissioning processes of tier two services prevents sustainable and innovative weight management practice which is detrimental to patients, falls short of addressing health inequalities and negatively impacts staff health and wellbeing.

## 1 Introduction

Overweight and obesity are a global health epidemic, with the number of individuals living with the disease having grown rapidly over the last five decades (1) despite increasing interest in obesity interventions (2, 3). In England alone, 63.8% of adults are living with overweight or obesity (4); this high prevalence is not uncommon across the world and is predicted to continue rising over the next decade (5). Many attempts have been made to address rising levels of overweight and obesity, but interventions largely fail to produce significant short-term weight loss or maintenance of weight loss over the longer-term (6, 7). This is of particular concern due to the associated physical and mental comorbid diseases (e.g., type two diabetes, coronary heart disease), and wider social and financial implications, placing greater emphasis on identifying routes to support weight loss and healthy weight (1, 8, 9).

Weight management in the United Kingdom is currently based on a four-tier system, including general, population-level preventative messages (tier one; e.g., five-a-day fruit and vegetable campaign, physical activity guidelines), community-based approaches (tier two; e.g., commercial weight loss groups), multidisciplinary specialist healthcare for complex weight management issues (tier three), and the provision of bariatric surgery (tier four) (9). Despite concerns around the growing challenges, obesity and weight management services are not mandatory functions of public health teams within local authorities (LAs; local government responsible for a range of services, including health and social care) in the UK. Instead, The Health and Social Care Act 2012 (10) mandates that public health provisions are available for child health (including the National Child Measurement Programme), sexual health (e.g., testing for sexually transmitted diseases), quinquennial health checks for those aged 40 to 74 years, and emergency preparedness. Service commissioners within LAs are required to make decisions on service provision which align to mandatory functions, but also address the health issues most relevant to their local communities (11), all on ever restricted budgets (12). This often means non-mandatory functions—such as weight management—are not prioritised or funded within LAs, with inequity in service provision and funding allocation between LAs (13).

The UK government has developed a number of policies over the last 30 years to address increasing levels of obesity (3). The latest policy, ‘*Tackling obesity: empowering adults and children to live healthier lives’* (14) aims to address overweight and obesity through the expansion of weight management services to provide greater support for individuals to manage their weight. This includes the expansion of tier two behavioural weight management services provided by LAs across England. To support this, in March 2021 the UK government announced £100 million of additional funding for weight management provisions (15), more than doubling the funding allocation for obesity services (16). Of this additional funding, £30.5 million was split across LAs in England specifically to support the expansion of tier two services for adults (17). Yorkshire and the Humber, a region in the north of England, comprises of 15 LAs with rates of obesity in adults ranging from 59.5 to 76.5% (18). Across this region, LAs received a share of £4.2 million to expand their tier two services (17). However, it was later announced that the government would pause the provision of funding for the 2022/23 financial year (19) as “...the costs of the ‘living with covid’ plan had to be paid from existing department funding, so the money for healthy weight activities in 2022/23 had to be cut.” (20).

It is clear that obesity is a disease of inequality, resulting from systemic health inequity that disproportionately affects the most deprived and underserved communities (21). Therefore, obesity treatment requires greater focus on ‘levelling up’ and delivering health equity (22). As such, it is important to consider a more consistent, wider scale approach to addressing underserved communities who face challenges in managing their weight and are often unable to access support (8, 23). Over recent years, there has been increased focus on such underserved communities within healthcare and funding provisions (24). Indeed, where there is greater emphasis on recruiting underserved communities to tier two weight management services, there appears to be some success, with a recent evaluation showing 44% of enrolled users were from high-risk groups—specifically those who live in the most deprived areas of England, those from a Black, Asian or minority ethnic group, those with a mental illness, or those with a physical or learning disability (25).

It is important to consider how LAs are utilising funding allocation to address overweight and obesity as well as inequalities in weight management services. While localised *ad hoc* evaluation of additional funding use has been carried out within Yorkshire and the Humber, there is no combined evaluation to establish best practice and/or shared learning. This is particularly pertinent given some services remain and new services may be developed should funding be made available in the future. To maximise the benefit from work of service commissioners and providers, the present work aimed to: (i) collect and collate learning and best practice; (ii) consolidate learning and provide guidelines/recommendations to aid rapid redeployment should funding return in subsequent financial years; (iii) identify whether key drivers of success for reducing inequality in public health interventions are also applicable to weight management.

## 2 Methods

### 2.1 Study design

The present qualitative study involved semi-structured interviews and a co-production workshop. A critical realist paradigm was applied as this maps well to systems thinking (26). This paradigm allows researcher reflexivity of social reality, and the reasoning, motivation and intention of individuals (27); within a weight management context, this allows reflection on the observable (e.g., service provision) and unobservable factors (e.g., systemic inequalities) impacting service outcomes. This also allows data to be participant-driven; the commissioner group are viewed as experts, leading the narrative and recommendation generation, with researchers actively reducing any preconceived ideas. The stakeholder framework development event embraced research reflexivity by allowing stakeholders to discuss, prioritise and amend recommendations that were generated by the qualitative research interviews. Reporting follows the Consolidation Criteria for Reporting Qualitative Research (COREQ) checklist (28)(see Supplementary material).

### 2.2 Researcher positionality

JB (RNutr) is a White British male lecturer and researcher, specialising in the fields of obesity, weight management, eating behaviour and appetite. He holds a PhD in Nutrition and has expertise in mixed methods research. EI (ANutr) is a female British-Cypriot PhD student and graduate teaching assistant with a background in sports science and experience of quantitative and qualitative methods. Her PhD focuses on optimising physical activity after gestational diabetes using realist inspired methods. KH (MCIPS Chartered) is a South Asian female and holds a MSc in Logistics and Supply Chains Management, with experience across engineering, events and inventory management, diversity and inclusion, and food and nutrition disciplines. NE, a Black African female, holds a MSc in Public Health with experience of mixed-methods approaches focussing on health equity, inequality and disparities. KH and NE were Masters students at the time of data collection and analysis and employed as research assistants. NC, a White British female, is a health and wellbeing programme manager within the OHID Yorkshire and the Humber regional team. She holds a Masters degree in Public Health Promotion and leads on the topics areas of obesity, physical activity and health and work. LN (RD, RNutr) is a White British female senior lecturer and researcher who is currently studying for a PhD in obesity systems and inequality. She has experience of mixed methods research, specialising in the fields of weight management, diabetes, systems evaluation, co-production and health inequalities. The authors work closely with voluntary, community and social enterprise organisations and wider healthcare systems.

### 2.3 Participants

Purposive sampling was used to recruit weight management service commissioners within Yorkshire and the Humber. Participants were recruited via the Office for Health Improvement and Disparities (OHID), with the study aiming to recruit representatives from all 15 local authorities. Commissioners were invited to participate in an individual one-hour online semi-structured interview to discuss their perspectives of tier two weight management service development, design, provision, success and best practice. All procedures were approved by the Sheffield Hallam University research ethics committee (ID: ER46148148). Participants were provided with an information sheet and completed informed consent procedures prior to the interview.

### 2.4 Interview schedule

An interview schedule was developed in line with the Public Health England (29) standard evaluation framework for weight management interventions to allow for a standardised data collection under three mains areas; service provision, service users, and service outcomes and evaluation. This also ensured that best practice evaluation recommendations were considered. Interviews were conducted by one author (from among JB, KH, NE) and recorded via Zoom. Transcripts were produced using Otter.ai, with all transcripts checked for errors and verified by one author (JB). Comments were anonymised, removing the names of individuals, companies, and local authorities. A copy of the interview schedule is available in the Supplementary material.

### 2.5 Framework analysis

A framework analysis was designed based on the framework proposed by Davey et al. (30) which consists of five principles for reducing health inequalities: (i) healthy-by-default and easy to use initiatives; (ii) long-term, multi-sector action; (iii) locally designed focus; (iv) targeting disadvantaged communities; and (v) matching of resources to need. Transcripts were read through to familiarise and immerse the authors with the content. Each transcript was independently analysed by two authors (from among KH, NE, JB, LN or EI) who extracted quotes and then analysed using framework analysis (31, 32) with discrepancies resolved through discussion. Data are reported in line with this framework and supported by anonymised quotations.

### 2.6 Stakeholder engagement event

Following analysis of the semi-structured interviews, an in-person workshop event was held in November 2023. Participants were public health service commissioners (*n* = 7) and the compassionate approach lead from one LA. A compassionate approach focusses on health gains and accounts for the wider context of an individual’s lives and aims to address weight without stigma or judgement. The research team provided an overview of findings from the interviews and request further input; participants were asked to ‘sense-check’ the findings presented, and to add additional context or information where they felt this was important. The participants then co-produced and prioritised recommendations to improve implementation of new or existing tier two weight management provision. Participants were split into two groups and guided by the researchers through a series of questions to establish what is currently working well, what could be done better, who the key actors and stakeholders in the system are for weight management services, and what recommendations they would give to the stakeholders and system to achieve appropriate, evidence-based weight management provision which addresses inequality. At the end of the session, the participants were asked to review a series of recommendations that had been generated by the research team from the synthesis of qualitative interview data, and develop them by adding their own suggestions, before prioritising the set of recommendations. Recommendations were agreed and prioritised by the participant group, independent of the research team. No formal analysis was conducted following this event; recommendations as outlined by the participants are included in section 3.6.

## 3 Results

A total of 11 commissioners representing nine of the local authorities within Yorkshire and the Humber were interviewed between January and April 2023. An additional interview was conducted with a compassionate approach lead within one local authority to provide context on comments from the service commissioner. Six local authorities declined to participate due to lack of capacity (*n* = 4), the individual who commissioned the original service was no longer in post (*n* = 1), and no response (*n* = 1).

### 3.1 Principle 1: Healthy-by-default and easy to use initiatives

This principle focuses on those factors that change the conditions in which people work and live to make “health-positive choices” (30). Within the interviews, the concept of healthy-by-default was well recognised and understood to be important but this was not reflected “on the ground” in current public health work for obesity and weight management.

*“…it’s a tricky one, isn’t it? Because we know that it’s more than just weight management services. It’s, it’s much bigger, it’s wider determinants. It’s, it’s everything, isn’t it?”* (LA3).

*“Because a tier two programme focuses on individuals as if it’s, it’s their fault that they are living with overweight or obesity. …it’s not… We’re in a cost-of-living crisis. …we live in an environment that’s obesogenic…people can go through a tier two programme and learn…about how to live and lead a healthier lifestyle. But they are still just going to be chucked back out of this programme into an environment that is not great.”* (LA6).

Commissioners and public health workers were aware of the need for whole systems approaches which would provide easier access to affordable, healthier food and improved opportunity for physical activity and were starting to embed whole systems thinking into their public health strategies.

*“It’s a whole partnership strategy around supporting people with physical activity, and food and things. So broader kind of whole systems obesity approach.”* (LA9).

However, in lieu of whole systems changes, they found that other factors in their interventions had been successful for improving recruitment and engagement such as: making the service free at the point of access; allowing choice of service, and at a time and place (be that online or face-to-face) which suited the service user; allowing access to services when the service user felt ready to engage; targeting underserved groups who were traditionally underrepresented in the service offer; changing the focus from weight loss to a wider and more holistic range of outcomes, including enjoyment of and engagement with the service. This was evidenced in some of the service outcomes which demonstrated the impact of these services.

*“I think, for us, the inclusion of people with learning disabilities is not great. …But the the, the feedback we got from the users and the carers that came, was just how how much that meant to those, those people. And I think that’s a [sic] really amazing, you know, we had good weight loss. But I think more more for me, how they felt included, how they felt welcomed, how they came every week, how that was something they looked forward to, that’s a real positive. And that’s something that’s, that’s really key.”* (LA7).

While the commissioners acknowledged that quantifiable outcomes – largely focussing on weight loss – are required to measure using traditional parameters of service success, they felt there was a mismatch between these success metrics and the concept of healthy-by-default. Rather than focussing on weight loss, commissioners wish to incorporate more holistic and individual measures of success.

*“Obviously, it being a weight management programme, the whole thing was around people losing weight. Erm from a personal view, that’s that that is a sticking point for me. Yes, I know, we need to get people to have a healthy weight. However, I’m not sure our weight management programme is the way to do that. That’s my personal opinion. So for me, it was more about how to get people engaged and enjoying being more active. How do we get people to have those conversations and be a little bit more considerate about what they are eating? Rather than, ‘Oh, we have got hundreds of people that have lost 5% of weight’, because really what does that mean? So once they finish the programme, have they learned how they made behaviour change? Did they enjoy what they were doing? Are they able to continue it? That for me is much more important than losing weight. But in terms of this because of the, because of the way the funding was, our primary goal was to get people through your programme, pick up the data and make sure there was some weight loss at the end of it.”* (LA7).

In one LA, there was particular interest in embedding the compassionate approach to weight within both service provision and measures of service success.

*“But we decided the compassionate approach would be the thread throughout so that although people have to be weighed on the programme, which is part of the [NICE] guidance, we did not want the emphasis to be on the weight. We wanted it to be on people feeling good, hence the name [redacted: local authority service]. And the compassionate side of reducing the stigma. And actually, some of the feedback we have got through the compassionate team is that came out quite strongly that people liked that.”* (LA3).

When initiatives are easy-to-engage with and provide the resources for individuals to access and enact health-promoting behaviours, this is likely to have the biggest impact on the reduction of health inequalities (30). Therefore, it is of paramount importance that this is the focus for LAs, but it will require a huge amount of work across multiple sectors to achieve such an environment.

### 3.2 Principle 2: Long-term, multi-sector action

Interventions need to be multi-sector, multi-level and long-term to achieve a sustainable impact on health, wellbeing and reduction of inequality; an intervention which focuses solely on one measure or determinant of health is unlikely to be of adequate value (33). This was well recognised by the commissioners and work was underway to engage the ‘Health in All Policies’ (34) approach to improve population health and health equity.

*“…we recognise that weight management is one small part of that whole obesity approach. Erm, and, you know, I don't think for one second that weight management is the answer, because it isn't the single solution. But it's very much part of the solution. …we're looking very holistically across what the council commissions in terms of children and families, erm that kind of the whole policy agenda around, you know, high sugar, high fat stuff. And, you know, the local declaration on healthy weight, all of those things, collectively and collaboratively trying to think about actually, what can we do as a partnership and an alliance moving forward acknowledging that, you know, we’re one small piece of the puzzle?”* (LA1).

Practical approaches were being taken which improved engagement with (and therefore referrals from) general practitioners (GPs), primary care professionals and social prescribers. Many local community, place-based organisations were involved with the provision of additional activities although often at additional cost.

The short-term funding allocation, bureaucracy associated with spending and procuring appropriate services and set-up time, combined with the abrupt end to funding provision resulted in short-term projects and interventions which were delivered at cost to the public purse, but in a manner which was unhelpful to the communities that LAs were trying to serve.

*“We didn't have time to do any research beforehand. …I'd have changed the, I'd have had a six month run in, so would have had six months to say, 'Okay, we're going to ask for this service', and you would do it in all the ways where it would be, you would go out to you got to tender, you would do the research beforehand, you would probably do some research within the local area to find out what would be acceptable you would do it all in that way, on the way that we're taught to do it in theory. …So because of the short timescales, we basically used our knowledge and experience to set it up.”* (LA5).

*“And we know that it [behaviour change] takes time. And you know, a six-month programme is not going to help is it because that's what it became, in the end, it wasn't 12 months, it became more six months.”* (LA3).

LAs were put under pressure to deliver short-term services by the commissioning process and to develop and deliver these services at speed and within the constraints of existing mechanisms which led to some programmes not being delivered in a way that the commissioners would ideally choose. However, where services were ‘piggy-backed’ onto existing services or extended from existing provision, there were more success stories with one LA stating, *“I think we have a robust, sustainable programme.”* (LA4).

### 3.3 Principle 3: Locally designed focus

The LAs often focussed on locally designed and tailored services, some of which were data driven. By understanding those who currently used and accessed existing services, and identifying those populations and communities where there was a service need, commissioners utilised the additional funding to focus on and align with the needs of underserved groups within the local community.

*“We knew from the data, that there were certain groups that weren't accessing our local service. …ethnic minority communities or underserved groups, men and people with severe mental illness or SEND [special education needs and disabilities] communities. …so our main service that we already had, they essentially upskilled locally, local community groups that worked with those communities that I've just listed. And they put on their own programmes for those groups, with the aim to improve the uptake for those communities.”* (LA6).

There were also assumptions based on non-attendance or uptake of current service such as digital weight management provision, that the local referral pathways were lacking capacity, that the location of the service was suboptimal, and that the current service was not suitable for everyone.

*“Erm, and the previous tier two model, by the way, had always been done in the hospital and it failed the first time round.”* (LA3).

Where LAs had more time and capacity, they worked with local communities and stakeholders to understand the barriers to uptake of the service and tailor the service to the local need.

*“So the providers spent some time talking to stakeholders and communities about what might work for them. So for example, with learning disabilities, they talk to staff within the learning disabilities team who suggested that perhaps they given [sic] extended completion times and normally we suggest we have 12 weeks, they suggested maybe taking it to 18 weeks having a couple of breaks, they suggested repetition around subjects. And for those from, we had a group of Pakistani women and the providers made sure they had provision for translation. And also thought quite detailed about where and when to hold the sessions and what format they should be.”* (LA9).

Commissioners reported a conflict around how to best spend the money to *“…do the greatest good for the greatest number? Or do we do we do that targeted work with a much smaller number of people but actually have a bigger impact?”* (LA1). From a locality-focussed perspective this was often a huge challenge on a limited budget and for some they felt there was an inequitable service provision across their region.

*“But things like for example, [redacted: company name], which we know tends to be a little bit more popular with women, they have more coverage across the city in terms of location. So, if you did want a face-to-face group, and it narrowed your choices, depending on where you lived… So it left us with quite a patchy provision of coverage across the city.”* (LA2).

However, commissioners were very aware of the importance of locality and place-based models of care with some LAs feeling that their provision was already well-tailored to place, such as LA3: *“…we have a very strong locality themed model…”* Where programmes are tailored to place and community settings and embedded in, or develop community infrastructures, the reduction in inequalities is more likely (35). Best practice and learning should aim to use data to identify underserved populations, and to work with those groups and community assets to develop tailored, place-based services which manage the needs of the specific population groups (33).

### 3.4 Principle 4: Targeting disadvantaged communities

Commissioners reported they were successful in targeting the underserved and disadvantaged communities for which services were tailored and provided bespoke services to these groups.

*“So, I suppose the changes that came about from this funding were the development of self-referral options, so was open to a much greater number of people, those bespoke offers to specific client groups, i.e., learning disabilities and migrant communities. And again, obviously, the staff actually involved in the work actually being able to get out into the community and kind of reach a greater number of people really and promote the service.”* (LA1).

However, the services were not always co-designed with the underserved groups, and several LAs felt this was key to improving service provision in the future and enhance their learning to date.

*“I think we would definitely build on this, build on what we've learned. But, but spend some time consulting and seeing what people needed and seeing if those offers were out there for us to, to bring to the table in terms of targeting particular populations.”* (LA2).

It is well-evidenced that universal provision which does not specifically target underserved communities or account for their particular needs and barriers to health, are less effective in reducing, and may worsen, health inequalities (36–38). However, for some LAs, this targeted approach was not achievable, particularly where there was no existing tier two weight management service which could then be diversified.

*“I used to sit on the tier two meetings with other areas and listen with absolute fascination about what other areas were doing. And there were so many variations of this service and models. And some people are sitting there quite jealous, really, because they don't have the luxury of a basic service. And they've been able to add things on. And they've been able to target for example, learning disabilities, or particular older age groups, or particular, you know, hard, high-risk groups. But I didn't have that luxury. I literally just had to go at it as a universal service.”* (LA3).

For those who had capacity to target disadvantaged communities, different methods were used to achieve this. Some recruitment campaigns were targeted to the communities, while other LAs worked with community groups to develop a service which met the needs of a particular identified community.

*“We have some more targeted work where we actually work in, for example, the job centre, we’re based in for some of our more deprived wards”* (LA4).

Commissioners also invested in additional support to ensure equal and inclusive access to services, such as services in other languages or formats (e.g., Urdu, British Sign Language) and targeting specific cohorts. Additionally, services were situated within areas of deprivation to provide ease-of-access for individuals.

*“...it doesn't differ massively other than there is that focus on some specific cohorts. So, for example, we did use some of the funding to work with a group of people with autism. Similarly, we run a bespoke group for the migrant community. Because again, that was something that we just never had capacity to do previously.”* (LA1).

Some of the shared learning also pertained to the engagement with local community, voluntary and faith organisations and providers who already had well-established, trusted contacts in the communities. By promoting services through these trusted community connections, engagement and uptake of services was improved. Trust of voluntary, community and social enterprise organisations has been evidenced as important in previous literature (39).

### 3.5 Principle 5: Matching of resources to need

In context of the present work, “resource” refers largely to financial resources linked with the funding allocation, but also includes time and effort by commissioners to develop and roll out services. As discussed above, commissioners reported conflict around how to best to utilise the funding, and whether they should aim for a bigger impact in a smaller focussed population, or provide a broader, more generic service which can be accessed more widely by larger numbers of individuals. From a locality-focussed perspective this was often a huge challenge on a limited budget and for some they felt there was an inequitable service provision across their region.

*“I think one thing that we've definitely recognised is because of the need locally, erm, clearly, our existing funding that we have is not going to even scratch the surface in terms of meeting the need. So, I think we need to find some of those alternatives.”* (LA1).

However, evidence suggests there is better return on investment where funding is spent in deprived areas (40, 41), and therefore targeting specific underserved communities is likely to be a better way to spend money from the public purse, but this is counteracted by LAs who need to guarantee they have “*got enough demand to justify the costs”* (LA8). Commissioners also found the restrictions placed on how funding could be spent, as well as time pressures, impacted the quality of their service and their ability to match resource to need.

*“And it was also all the bureaucracy, [redacted: name of individual], you know, because in a council it's nothing straightforward. Everything's got to be done. You know, every i dotted and every t crossed. …We did, obviously, in the spec, try to target the more at-risk groups. And we have got, they did present data that, you know, they have reached some of the sorts of risk groups. But in the time period we had, we couldn't really test that out very well.”* (LA3).

Where LAs were able to consult with stakeholders and communities from their target groups they were also able to tailor services, often in simple ways, to capture the needs of these groups. As a result, this improved how effective the service was, and was therefore viewed as better value-for-money due to the increased engagement and service suitability. Funding was also allocated to help overcome some of the common practical barriers to attendance such as childcare provision or transport. Other services were delivered outside of standard workday hours to encourage attendance, or alongside employers’ wellbeing services which allowed employees to take time during their workday to engage with the weight management provision. Additionally, there were staff training needs which were identified by many commissioners and resource was allocated to help upskill the workforce to deliver effective and diverse programmes to support underserved communities. Training resource needs included cultural awareness and adaptations, language courses, easy read training for design and development of resources for managing mental ill health and suicidal ideation.

*“I think investing in training for the providers as well to accommodate different needs and resources that they, they would need. …I think it's investing in the teams to be able to accommodate individual needs of those clients. I mean, they're, they're brilliant, our providers are brilliant, they're really well experienced. And it's a real person-centred approach. But it's just broadening their skill set, I think, to accommodate what we see as people coming through with quite complex needs.”* (LA9).

However, a clear message which came out of this principle was the challenge of matching the resources to the needs of underserved populations, at speed, with minimal staff resource and budget and while addressing the complexity of the targeted participants. As a result, many interviewees described the emotional and mental health impact it had on their wellbeing, which meant it was particularly difficult when funding was suddenly withdrawn a short time afterwards.

### 3.6 Co-production of recommendations and guidelines

**Figure 1:**
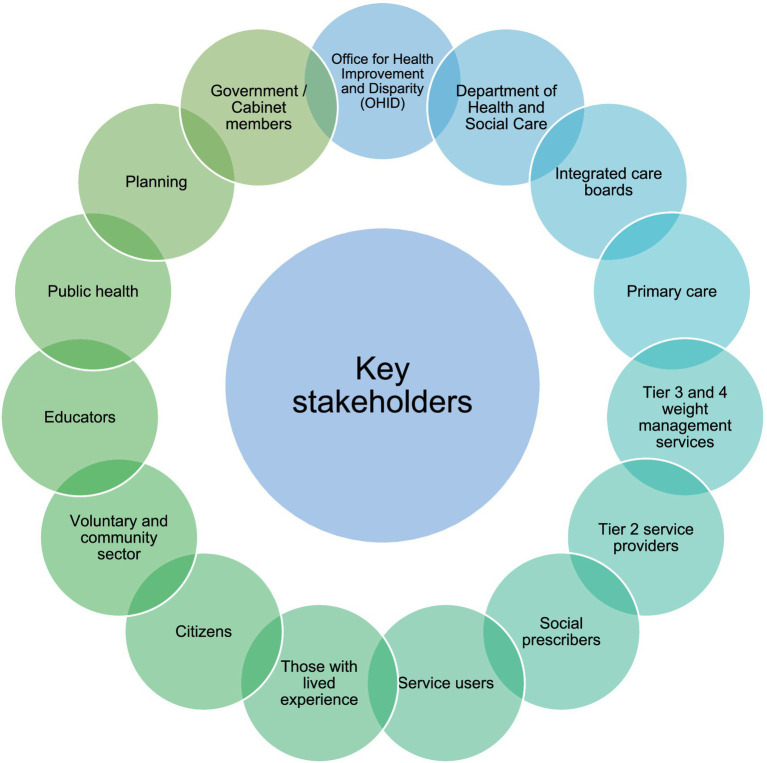
Key stakeholders in weight management within the UK identified by service commissioners.

The participants co-developed a set of 10 recommendations which they felt were representative of their views, knowledge and experience. The service commissioners recognised that for systems change to be successful and sustainable it needs to be multi-factorial, engaging a wide range of stakeholders (Figure 1), and therefore found prioritisation of recommendations challenging due to the nature of the obesity eco-system. Recommendations were therefore split into three levels: complexity of obesity; need for long-term support; need for practical solutions.

#### 3.6.1 Recommendations to address complexity of obesity

The first level of recommendations recognise that obesity is complex, and that the wider obesity system must be defined, recognised and engaged with at an early stage of intervention planning to ensure that weight management services are addressing an appropriate gap in the weight management context. As such, limited resources should be targeted to those most in need and early prevention of obesity, particularly in early years, should be key. Finally, being brave, innovative and “trying something new” (such as establishing alternative measure of success) was defined as a fundamental outcome for weight management services to be more meaningful to holistic wellbeing outcomes (Table 1).

**Table 1:** Recommendations on acknowledging the complexity of obesity and weight management.

Be brave	Be bold and innovative. Lessen restrictive funding parameters to allow commissioners to do things differently.
Consider alternative measures of success	Stop focussing on single outcome measures (e.g., weight). Being honest and realistic about weight loss expectations (e.g., likelihood of plateau or weight regain). Move towards a more compassionate approach. More focus on holistic and person-centred outcomes. Focus on psychosocial elements (e.g., self-care, self-efficacy).
Target limited resources to those most in need	Understand who is currently engaging in the service(s). Aim for early prevention (i.e., early years) and consider gaps in provision.
Recognise weight management is part of a bigger system	Whole systems approaches are required which actively engage key stakeholders. Health in All Policies approach. Create an environment for healthy choices. Explore impact of environment on health and weight. Consider efficacy of weight management services that focus solely on food and physical activity behaviours. Educate stakeholders on the complexity of obesity.

#### 3.6.2 Recommendations to address the need for long-term support

The second group of recommendations highlighted the importance of long-term support for participants attending services, using co-design with service users and stakeholders to develop services, recognising and addressing the complexity of the service user profile and ensuring the correct language is used for optimising recruitment and engagement with interventions and services (Table 2).

**Table 2:** Recommendations around long-term support in weight management services.

Co-design services with stakeholders	Co-design with key stakeholders and community members from underserved groups.
Recognise the potential complexity of participants	Consider the training needs of the workforce and provide resources to allow staff to upskill and adapt materials and content to meet those needs. Provide more inclusive, accessible services.
Consider the language that is used	Replace ‘weight management’ terminology with more inclusive language with a focus on ‘health’ and ‘wellbeing’. Avoid language which promotes weight stigma.
Provide long-term support (3+ years)	Make the commitment and make it long-term. Focus on long-term behaviour change. Lobby for longer term funding. More funding to support staff training to meet needs of specific communities.

#### 3.6.3 Recommendations to address the need for practical solutions

Finally, the third layer of recommendations highlighted the need for practical solutions for monitoring and evaluating services, using large data collected within the services, and to tailor communication and marketing of services effectively to appropriate populations (Table 3).

**Table 3:** Recommendations on data usage and communication.

Use datasets to monitor and evaluate services	Use the data collected and knowledge generated and learn from trial-and-error to adapt services ‘in action’. Invest more in evidence and accurate data collection. Consider systems for reporting.
Invest in communications and marketing	Adapt and be specific to different population groups. Consider messaging to general practitioners and other healthcare professionals to ensure appropriate referrals.

## 4 Discussion

This work specifically looked to identify how additional funding was used by LAs, explore how target populations were identified and how services were tailored, and identify recommendations for the provision of future tier two services. Data from the present study suggests the main benefit of the evaluated interventions had been the capacity to test new ideas and processes and to evaluate the learning. There were many key learnings which had been shared in the interviews and the workshop which have developed into recommendations for practice. However, this was in the context of constraints and limitations. It was therefore challenging for LAs to overcome barriers to innovative practice, and this prevented the whole systems approach work and delivery of interventions which fully addressed all five of the recommendations outlined in the framework proposed by Davey et al. (30). As a result, commissioners and service providers were often knowingly doing “the wrong thing well.”

The management of obesity is changing, with an increased focus on whole systems approaches to weight management and addressing health inequalities caused by the social determinants of health (42–44). Recruitment and retention for weight management services is notoriously challenging (45, 46), and the access to and engagement with healthcare is variable and dependent on sociocultural and socioeconomic status (47). While adherence to programmes may be improved through early weight loss success and more advantageous baseline characteristics (e.g., lower body mass index [BMI], better overall mood), many barriers to engagement exist (e.g., perceived lack of time, perceived lack of knowledge, social pressure, poor physical and mental health, socioeconomic constraints, lack of enjoyment in exercise) (48).

While it remains important to address obesity on a population level, there is increasing concern for underserved communities (8, 23). It is important to recognise that those often participating in weight management services are unlikely to be representative of the diverse population the services aim to serve. The typical demographic of an individual accessing weight management services in England is a middle aged, white, heterosexual female without any disability (49). However, overweight and obesity are more prevalent in wider demographic groups. For example, there is higher prevalence in those with disabilities, people in Black ethnic groups, those with no or little education, or those who live in the most deprived regions (4). Overall, commissioners and LAs had very good population insight, experience-led local knowledge, and were striving to work in a whole system, proportionate universalism model which was evidence-based and would lead to reductions in health inequality. However, the reality of what could be achieved was constrained due to commissioning processes, bureaucracy and short timescales, in addition to the national guidelines and policy which focussed on weight change outcomes (9) and therefore reduced the efforts of some of the compassionate and health-focussed approaches. The short-term funding prevented patient and public involvement consultation work, curtailed the test-and-learn process and prevented sustainable practices. As a result, staff wellbeing suffered with both commissioners and service delivery teams reporting issues from the pressure of organising an intervention at speed, the disappointment of reducing service activity quickly with perceived limited benefit to the communities they serve, and concerns over job security for service delivery employees. This is evidenced in employment literature, with a clear association between job insecurity, more organisational change and poorer wellbeing (50, 51).

Despite weight management services not being a mandatory function of public health (10), following the pause of funding, 12 of the 15 LAs provided tier two weight management services in the Yorkshire and Humber region. However, there were profound differences in service availability within a LA and the contrast in provision between LAs was even starker with some commissioners reporting no baseline service provision at all. This suggests a ‘postcode lottery’ which may lead to inequality within and between regions. Indeed, there were concerns regarding inconsistent and patchy services; in one LA, referrals which did not fit the rigid funding inclusion criteria meant such referrals were rejected, for example because the individual’s body mass index was not high enough (i.e., motivated individuals who were seeking support would need to gain more weight before they were eligible to access help). This demonstrates that strict commissioning guidelines prevent services from matching resources to need leading in inequity in access, engagement and retention within current weight management interventions. Even ‘best practice’ and multidisciplinary models based on National Institute for Health and Care Excellence (9) guidelines have limited success (52).

Knowledge of place-based issues within the commissioner group was very good, and their reference to deprivation, inequalities and wider determinants of health was consistent throughout the interviews. There was also recognition that delivery of a tier two weight management service was not the answer to systems change or tackling obesity more broadly. LAs were aware of their underserved populations and primarily used data-driven approaches to identify these groups. However, organising specific provision for each group on a proportion universalism approach and with a limited budget was challenging. As a result, co-production with local populations and stakeholders was limited, but where this had been achieved, the service design and development had successfully overcome barriers to attendance and engagement. It is therefore important to capture the voices of those underserved communities prior to commissioning and service design and delivery (53). A way of successfully managing this, given the limited resources, is for LAs and commissioners to work closely with the voluntary, community and social enterprise sector to access local, marginalised voices through these partnerships. Additional best practice would include the development of patient and public involvement initiatives (33).

Stability of service provision for adult weight management services has been compromised due to diverted budgets and the cost-of-living crisis (19, 20). Abrupt withdrawal of or inconsistent funding can have negative impact on health outcomes, including increased weight status of individuals who would otherwise have had access to services, with similar cases previously analysed [e.g., Mason et al. (54)]. Cutting funding likely places additional stress on already strained services and the UK government have been criticised regarding their funding of weight management and wider obesity-related services (23).

## 5 Strengths and limitations

The present work was timely and placed the service commissioners—as the experts—at the heart. As a result of this, the work had significant support from service commissioners and OHID with response to the research and development of recommendations. Additionally, this work had good representation from LAs across the Yorkshire and Humber region as well as different service types and commissioning priorities. Despite differences in service design, priorities were recognised and agreed by all stakeholder. A limitation of the current work is the lack of service user and provider involvement. While this was not within the original focus of the present study, having such involvement would provide important context the findings, particularly linked with the five key principles discussed above. In addition, we discuss inequality within a weight management context; measuring inequalities within a system is difficult, and no measure was used in the present work. Future work should explore how inequalities can be quantified within weight management services.

## 6 Conclusion

The present work acknowledges that targeted weight management services are only one small piece of the puzzle, and that this is widely recognised. Therefore, recommendations include targeting underserved communities, focussing on early prevention, addressing weight management in a whole systems context, and embracing innovative and holistic approaches to weight management. Current short-term funding and restrictive commissioning processes of tier two services prevents sustainable and innovative weight management practice which is detrimental to patients, falls short of addressing health inequalities and negatively impacts staff health and wellbeing. While this research project provides a process evaluation of tier two weight management commissioning, the insight and learning are common to many commissioning processes and the recommendations can be applied to a broader public health context.

## Data availability statement

The raw data supporting the conclusions of this article will be made available by the authors, without undue reservation.

## Ethics statement

The studies involving humans were approved by the Sheffield Hallam University research ethics committee (ID: ER46148148). The studies were conducted in accordance with the local legislation and institutional requirements. The participants provided their written informed consent to participate in this study.

## Author contributions

JB: Writing – review & editing, Writing – original draft, Visualization, Validation, Supervision, Project administration, Methodology, Investigation, Funding acquisition, Formal analysis, Data curation, Conceptualization. EI: Writing – review & editing, Investigation, Formal analysis, Data curation. KH: Writing – review & editing, Investigation. NE: Writing – review & editing, Investigation. NC: Writing – review & editing, Resources, Conceptualization. LN: Writing – review & editing, Writing – original draft, Validation, Supervision, Project administration, Methodology, Funding acquisition, Formal analysis, Conceptualization.
