# Supplementary material for: “We’re one small piece of the puzzle”: evaluating the impact of short-term funding for tier two weight management services
Source: Front Public Health. 2024 May 22;12:1381079. doi: 10.3389/fpubh.2024.1381079 (PMC11150676; doi:10.3389/fpubh.2024.1381079)
Supplement: Supplementary file 1 [file Table_1.DOCX]

**SUPPLEMENTAL MATERIAL**

**Tier 2 weight management services in Yorkshire and the Humber: Evaluating the impact of additional government funding.**

Jordan D. Beaumont^a,*^, Elysa Ioannou^b^, Krishna Harish^a^, Nnedinma Elewendu^c^, Nicola Corrigan^d^, Lucie Nield^a^

*^a^ College of Business, Technology and Engineering, Sheffield Hallam University, Sheffield, S1 1WB, UK*

*^b^ Sport and Physical Activity Research Centre, Sheffield Hallam University, Sheffield S10 2BP, UK*

*^c^ College of Social Sciences and Arts, Sheffield Hallam University, Sheffield, S1 1WB, UK*

*^d^ Office for Health Improvement and Disparities, Department of Health and Social Care, Blenheim House, Leeds, LS1 4PL, UK*

** Corresponding author, email:* [*j.beaumont@shu.ac.uk*](mailto:j.beaumont@shu.ac.uk)

**Consolidation Criteria for Reporting Qualitative Research (COREQ) Checklist**

Adapted from: Tong A, Sainsbury P, Craig J. Consolidated criteria for reporting qualitative research (COREQ): a 32-item checklist for interviews and focus groups. *International Journal for Quality in Health Care*. 2007. Volume 19, Number 6: pp. 349 – 357

| **Topic** | **Item** | **Guide Questions/Description** | **Reported on Page No.** |
| --- | --- | --- | --- |
| **Domain 1: Research team and reflexivity** | | | |
| *Personal characteristics* | | | |
| Interviewer/facilitator | 1 | Which author/s conducted the interview or focus group? | 7 |
| Credentials | 2 | What were the researcher’s credentials (e.g., PhD, MD)? | 6-7 |
| Occupation | 3 | What was their occupation at the time of the study? | 6-7 |
| Gender | 4 | Was the researcher male or female? | 6-7 |
| Experience and training | 5 | What experience or training did the researcher have? | 6-7 |
| *Relationship with participants* | | | |
| Relationship established | 6 | Was a relationship established prior to study commencement? | 7 |
| Participant knowledge of the interviewer | 7 | What did the participant know about the researcher (e.g., personal goals, reasons for doing the research)? | Not reported |
| Interviewer characteristics | 8 | What characteristics were reported about the interviewer/facilitator (e.g., bias, assumptions, reasons and interests in the research topic)? | Not reported |
| **Domain 2: Study design** | | | |
| *Theoretical framework* | | | |
| Methodological orientation and theory | 9 | What methodological orientation was stated to underpin the study (e.g., grounded theory, discourse analysis, ethnography, phenomenology, content analysis)? | 5-6 |
| *Participant selection* | | | |
| Sampling | 10 | How were participants selected (e.g., purposive, convenience, consecutive, snowball)? | 7 |
| Method of approach | 11 | How were participants approached (e.g., face-to-face, telephone, mail, email)? | 7 |
| Sample size | 12 | How many participants were in the study? | 8-9 |
| Non-participation | 13 | How many people refused to participate or dropped out? Reasons? | 9 |
| *Setting* | | | |
| Setting of data collection | 14 | Where was the data collected (e.g., home, clinic, workspace)? | 7-8 |
| Presence of non-participants | 15 | Was anyone else present besides the participants and researchers? | N/A |
| Description of sample | 16 | What are the important characteristics of the sample (e.g., demographic, data, date)? | 8 |
| *Data collection* | | | |
| Interview guide | 17 | Were questions, prompts, guides provided by the authors? Was it pilot tested? | Supporting material |
| Repeat interviews | 18 | Were repeat interviews carried out? If yes, how many? | N/A |
| Audio/visual recording | 19 | Did the research use audio or visual recording to collect the data? | 7 |
| Field notes | 20 | Were field notes made during and/or after the interview or focus group? | N/A |
| Duration | 21 | What was the duration of the interview or focus group? | 7 |
| Data saturation | 22 | Was data saturation discussed? | N/A |
| Transcripts returned | 23 | Were transcripts returned to participants for comment and/or correction? | N/A |
| **Domain 3: Analysis and findings** | | | |
| *Data analysis* | | | |
| Number of data coders | 24 | How many data coders coded the data? | 7-8 |
| Description of the coding tree | 25 | Did authors provide a description of the coding tree? | 7-8 |
| Derivation of themes | 26 | Were themes identified in advance or derived from the data? | 7-8 |
| Software | 27 | What software, if applicable, was used to manage the data? | N/A |
| Participant checking | 28 | Did participants provide feedback on the findings? | 8-9 |
| *Reporting* | | | |
| Quotations presented | 29 | Were participant quotations presented to illustrate the themes/findings? Was each quotation identified (e.g., participant number)? | 9-20 |
| Data and findings consistent | 30 | Was there consistency between the data presented and the findings? | 9-24 |
| Clarity of major themes | 31 | Were major themes clearly presented in the findings? | N/A |
| Clarity of minor themes | 32 | Is there a description of diverse cases or discussion of minor themes? | N/A |

**Interview Schedule**

Key:

**X.FX:** = follow-up question

**X.PX:** = prompt

**NB:** = note for the interviewer

**Introduction**

Thank you for taking part in our study. Today we will be conducting an interview to capture the expanded tier 2 weight management service provisions that your team put in place using the uplift funding provided by the government in the 2021/22 financial year. We may touch on your wider tier 2 weight management services, but today we are particularly interested in how you spent the uplift funding. There are no right or wrong answers to any of the questions, so please share your genuine understanding. In line with our standard and ethical practice, all information you share will remain anonymous and no sensitive data will be shared beyond the research team.

**Service Provision**

1. To start, can you give me an overview of the service you set up with the uplift funding?

1.P1: Content – what is covered? Behaviour of focus (e.g., diet, physical activity)

1.P2: Individually tailored?

1.P3: Length of time and frequency users engage with service; Contact time between users and service

1.P4: Mode of delivery

e.g., consultation (e.g., with nutritionist/dietician), exercise class, healthy cooking class

online, telephone, app, in-person, self-help resources, etc.

individual or group

1.P7: Where is it delivered (GP surgery, community/leisure centres, gyms, etc.)?

1.P8: Access (across whole local authority area, or specific region?)

1.P9: Creator (commissioner or provider?)

1.P10: Length of time service is/was commissioned

1.F1: Was any follow-up following completion included in the service?

2. Why did you choose to focus on this specific service?

2.F1: What were the factors that contributed to this decision?

3. What was the rationale for this intervention?

3.P1: Evidence (scientific publications, NICE/NHS guidance, primary research by local authority, user data, health statistics, PHE’s standard evaluation framework etc.)

4. What was your aim with providing this service?

4.F1: Did this change as the service progressed?

5. How does this service compare with your usual service provisions?

5.P1: Repetition of current services for new population, or novel and targeted?

5.P2: Similarities and differences

6. How do you think this service compared with the provisions of other local authorities within Yorkshire and the Humber, and the wider UK?

7. Can you provide an overview of who the service provider was?

NB: We do not need specific details (e.g., name, contact information) here but looking for a description of the type of entity that provided the service.

7.P1: Competencies of service provider (e.g., background, expertise, training, qualifications)

7.F1: Was this a new or existing provider to you?

7.F2: (If a new provider) How did you identify this provider?

7.F3: Did the provider receive any incentives, such as payment based on attendance or results?

7.F4: Do you feel there are any conflicts of interest with your use of this provider?

**Service Users**

8. Who were your target population?

8.P1: Demographics (e.g., age, gender/sex, ethnicity, race, sexuality)

8.P2: Specific group targeted – underrepresented?

9. Why did you choose to target this population?

10. How did you recruit service users?

10.P1: Referral mode (self-referral, GP referral, etc.)

10.P2: Service promotion (healthcare setting, via leaflets/posters/social media, word-of-mouth, community leaders)

10.P3: Any incentives for GPs or others to refer to the service?

11. Were there criteria people had to fit to access the service?

11.P1: Demographics (i.e., restricted to target group, or open to anyone?)

11.P2: Weight status/BMI

11.P3: Prior weight management experience?

12. Can you provide an overview of the population who used the service?

NB: We’re looking for differences between target population and recruited population here (i.e., did they recruit their intended population?)

12.P1: Total number, and recruited versus finished, any cap on numbers?

12.P2: Demographics (e.g., age, gender/sex, ethnicity, race, sexuality)

12.P3: Specific group targeted – underrepresented?

13. Were you able to recruit the target population?

13.F1: (If indicate issues) Were there any barriers to engagement?

13.F2: (If indicate issues) Did you notice any dropout, and if so, why did this happen?

14. Did you conduct any participant and public involvement (PPI) activity whilst planning the service?

14.F1: Were any of the additional needs of your population considered?

14.F1.P1: Access needs, childcare, caring responsibilities religious/cultural restrictions, etc.

14.F2: Did you consider any of the wider health, lifestyle and status of the target population?

14.F2.P1: Co-morbidities, socioeconomic status, employment, housing, health literacy, etc.

15. Did users receive any incentives?

15.F1: (If they did) What were these incentives?

15.F2: (If they did) Was this to sign up for the service, for continuous engagement, or following successful completion?

15.F3: How do you think this impacted uptake and engagement?

16. Did the user incur any costs to participate?

16.P1: Identify these costs

16.F1: (If they did) Do you think this affected service usage?

**Service Outcomes and Evaluation**

17. What were the main outcomes you hoped to achieve with this service?

17.P1: Weight-based (e.g., mass, BMI, BMI-z)?

17.P2: Any wider outcomes (e.g., wellbeing, self-esteem, food consumption, physical activity level)

17.P3: Primary versus secondary outcomes

18. How did you measure and monitor these outcomes?

18.P1: Frequency of monitoring

18.P2: Quality assurance (e.g., spot-checks by external assessor, self-assessment checklists)

18.P3: Validated or ad-hoc measures?

19. Was the service delivered as planned?

19.P1: (If not) Explore why it did not go to plan

20. Do you believe you were successful in achieving the service aims and outcomes?

21. Has the service been evaluated?

21.F1: (If it has, and we do not have the evaluation output) Could you share the evaluation with us?

22. Would you consider the service was cost effective?

22.F1: Would you be able to share the cost per participant?

23. Were there any challenges or barriers you had to overcome when designing and implementing this service?

24. Other than funding, are there any other barriers to providing this service?

24.P1: Issues with service providers

24.P2: Issues with recruitment or user engagement

24.P3: Issues with achieving aims

25. Do you feel you received sufficient funding to effectively deliver the service?

25.P1: (If not) Why?

26. Did any unexpected outcomes or adverse events occur?

27. Were the service users provided with any data on their progress?

27.F1: (If they did) What data did they receive?

27.F2: (If they did) Was this at the end of their participation, or periodically throughout?

28. Did you receive any feedback from users, or did you capture user satisfaction?

28.F1: (If they did) Can you provide an overview of this feedback?

29. How do these outcomes align with your local authority’s focus on health?

30. Were participants invited to other interventions or services as a result of participating in the uplift service?

31. If you were to receive the funding again, would you take the same approach?

31.P1: Why???

31.P2: What would be the same?

31.P3: What would be done differently?

32. What are the main learnings or best practice you would like to share with other local authorities?

**Conclusion**

Thank you for taking the time to participate in this interview. Your responses have been very interesting and will provide useful data for our evaluation.

32. Do you have any further comments you would like to make that you feel were not captured by our questions?

33. Do you have any question?
